# Parents’ Perceptions of Their Children’s Engagement in a Consumer-Based Meditation Mobile App: Cross-Sectional Survey Study

**DOI:** 10.2196/24536

**Published:** 2020-11-13

**Authors:** Megan Puzia, Breanne Laird, Jeni Green, Jennifer Huberty

**Affiliations:** 1 Behavioral Research and Analytics LLC Salt Lake City, UT United States; 2 College of Health Solutions Arizona State University Phoenix, AZ United States

**Keywords:** mindfulness, meditation, children, mental health, sleep, mHealth

## Abstract

**Background:**

In the United States, nearly half (48%) of school-aged children experience sleep disturbance that results in less than the recommended sleep duration, which may negatively impact mental health and behavior. Mindfulness interventions may improve sleep and mental health in youth. However, there are gaps in the literature regarding how children (2-12 years) and adolescents (13-17 years) practice mindfulness and the extent to which they benefit from these practices.

**Objective:**

The purpose of this study was to determine parents’ perceptions of their children’s engagement with a consumer-based mindfulness meditation app and the extent to which they believe their children have benefitted from using the app, particularly with regard to sleep.

**Methods:**

This study is a secondary analysis of a cross-sectional survey in adult subscribers (N=11,108) to the mindfulness meditation mobile app Calm. Participants who indicated that they had a child or children younger than 18 years (2944/11,108) who used the Calm app were asked additional questions related to their perceptions of their children’s engagement with Calm. Descriptive statistics were used to assess children’s app engagement, and chi-square tests and binary logistic regression models were used to assess differences in children’s usage based on gender and age.

**Results:**

Among the survey respondents, approximately half of the parents (1537/2944, 52.21%) reported that their children used Calm. Children used Calm mostly for (1) sleep (1168/1537, 75.99%), (2) stress (491/1537, 31.95%), (3) depression or anxiety (430/1537, 27.98%), and (4) improvement of overall health (215/1537,13.99%). Older children were more likely to begin using Calm to reduce stress, depression, or anxiety, whereas younger children were more likely begin using Calm to improve sleep. Most children used Calm when lying down to go to bed (1113/1529, 72.79%). Children were most likely to use sleep stories at night (1144/1207, 94.78%), followed by music and soundscapes (749/1114, 67.24%), meditations (736/1120, 65.71%), and breathing exercises (610/1092, 55.86%). Nearly all parents believed that using sleep stories was helpful for their children’s sleep (1090/1128, 96.63%), and the majority of parents felt that the other components were also helpful for their children’s sleep (music and soundscapes [570/728, 78.30%], meditations [445/696, 63.94%], and breathing exercises [610/1092, 55.86%]).

**Conclusions:**

To our knowledge, this is the first study to explore parents’ perceptions of how their children or adolescents use a popular consumer-based mindfulness mobile app (ie, Calm). As the majority of children use the app for sleep, mindfulness meditation mobile apps should consider incorporating age-appropriate sleep content to meet the needs of this audience. More research is needed to confirm the feasibility and effectiveness of mindfulness meditation apps for improving sleep and mental health in children and adolescents.

## Introduction

In the United States, half (48%) of school-aged children (6-14 years) experience sleep disturbance (eg, insomnia, nightmares, sleep walking) most nights of the week that results in less than the recommended sleep duration (ie, 9 hours) [1,2], which is an important public health problem [3,4]. Sleep difficulties often persist from early and middle childhood through adolescence and early adulthood [5], and they are predictive of neurodevelopmental, psychosocial, and behavioral health concerns in later life [1,6]. There is a need for tools that have the potential to improve children’s sleep and downstream effects that poor sleep may have on emotional and behavioral outcomes.

Mindfulness is the practice of being intentionally engaged in the present moment with nonjudgmental awareness of one’s thoughts, feelings, and sensations [7]. Studies have suggested that mindfulness interventions can lead to improvements in stress, mental health, and sleep in both adults [8] and youth [9-11]. Although there is growing interest in mindfulness-based activities for children (2-12 years) and adolescents (13-17 years) [12], especially in schools [13], there are substantial knowledge gaps in the ways that children and adolescents practice mindfulness and the extent to which they benefit from these practices, particularly in regard to sleep.

The increasing use of technology (eg, smartphones, tablets) in children’s lives makes mobile apps a convenient way to deliver mindfulness-based interventions that support health and well-being [10]. Though the current evidence related to the engagement and efficacy of mindfulness-based mobile apps in children and adolescents is lacking, research suggests that mindfulness-based mobile apps may be beneficial for mental health in adolescents [14,15], and the online delivery of mindfulness interventions are often better attended or preferred than in-person delivery [15,16]. However, a recent review of 36 free mindfulness-based apps for children reported that most failed to achieve a good quality rating score [10]. There is clearly room for improvement in mindfulness apps for the benefit of children and adolescents. Considering the potential for mindfulness apps to help improve sleep and mental health, more research is needed to better understand how children and adolescents engage with popular mindfulness apps in order to better meet the needs of this audience. Therefore, the purpose of this study is to determine parents’ perceptions of their children’s engagement with a commercially available mindfulness-based app and the extent to which they believed their children benefitted from using the app, particularly with regard to sleep.

## Methods

### Overview

The findings presented in this paper were part of a survey conducted in adult subscribers to the mindfulness meditation app Calm (N=11,108) [17]. Subscribers were eligible for the survey if they (1) were at least 18 years old, (2) could read and understand English, and (3) had used at least one sleep component of the Calm app in the previous 90 days. Subscribers received an email inviting them to answer a survey regarding their use of Calm. Participants who had children younger than 18 years were also asked about whether and in what ways their child or children used Calm.

### Measures

An investigator-developed survey was used to obtain information from parents about how their children used Calm (see Textbox 1 for a complete list of questions).

**Survey questions for parents.**

**1. Do your children use Calm?**

- Yes

- No

**2. What are the gender(s) of your children who use Calm?**

- Boys

- Girls

- Other

**3. How old are your children who use Calm?**

**4. Why do your children use Calm?**

- Improve overall health

- Reduce stress

- Reduce depression or anxiety

- Improve sleep

- Other

**5. When do your children use Calm?**

- Within the 30 minutes after waking

- In the morning, but not within 30 minutes of waking

- In the afternoon

- In the evening

- At night, but not within 30 minutes of going to bed

- Within 30 minutes before lying down to go to bed at night

- While lying down to go to bed at night

- When I wake up during the night and I can’t fall back asleep

**6. Which best describes how your children use Calm at night?**

- I try to use Calm on a regular basis

- I sometimes/occasionally use Calm

- I use Calm only when I need it

**7. How many times per week do your children use each component at night or in order to help with sleep?**

- Sleep Stories

- Music/soundscapes

- Sleep meditations

- Meditations

- Breathing exercises

- None of the above

**8. [Of selected components] Do you think that using each of the following components has been helpful for your children's sleep?**

- Very much helpful

- Somewhat helpful

- Not noticeably helpful

### Statistical Analyses

All analyses were conducted in IBM SPSS Statistics version 26.0 (IBM Corp). Descriptive statistics were used to assess children’s app engagement. Differences in children’s usage based on gender and age were assessed using chi-square tests and binary logistic regression models.

## Results

Of the 11,108 survey respondents, 2944 (26.50%) reported having children younger than 18. Half of the parents (1537/2940, 52.28%) reported that their children used Calm (see Table 1).

**Figure 1:**
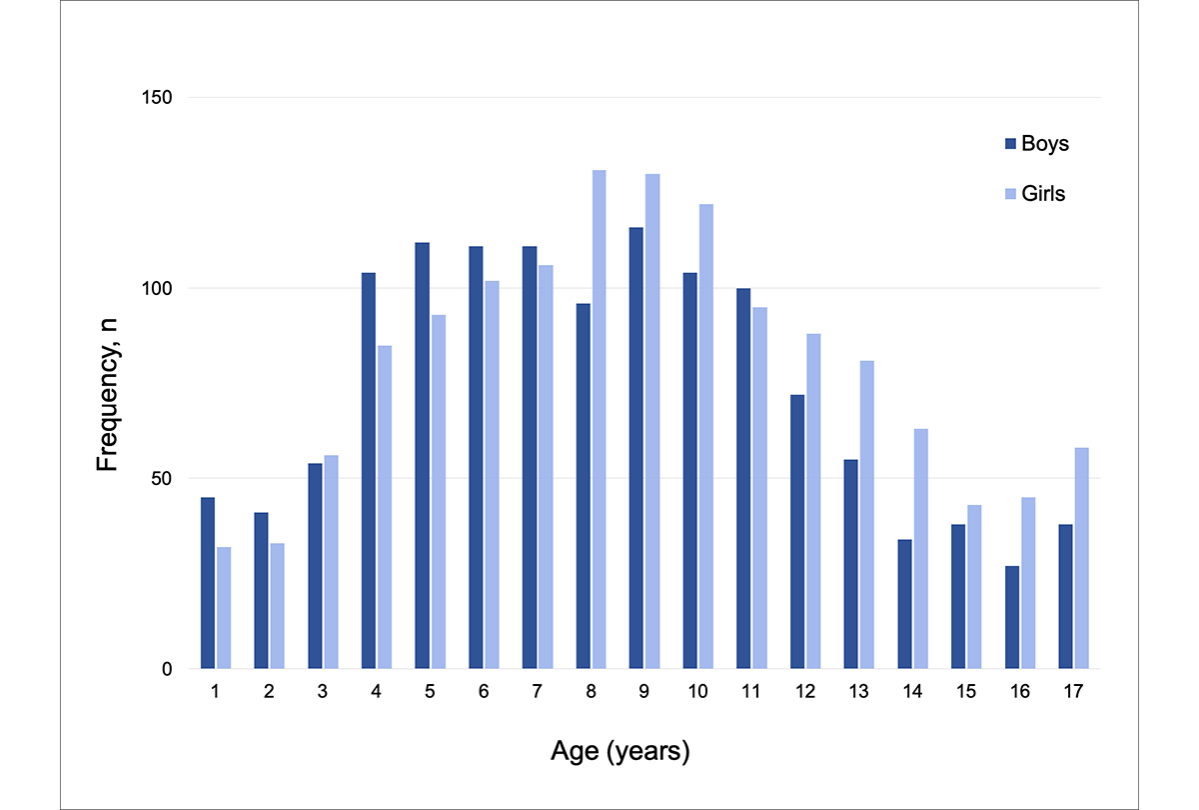
Age distribution of children using Calm.

On average, children who used Calm were aged 9.66 (SD 4.11) years (see Figure 1). Approximately 60.83% (935/1537) of parents had boys and 66.95% (1029/1537) had girls who used Calm. Boys using Calm tended to be younger than girls (*F*_1,710_=19.91; *P*<.001; *d*=0.34).

Parents most often reported that the reason that their children started using Calm was to improve sleep (1174/1537, 76.38%), followed by reducing stress (493/1537, 32.08%), reducing depression or anxiety (427/1537, 27.78%), and improving overall health (216/1537, 14.05%). Parents of older children were more likely to report that their children began using Calm to reduce stress, depression, or anxiety, whereas parents of younger children were more likely to report that their children began using Calm to improve sleep (see Table 2). There were no gender differences in the reasons for using Calm.

Most parents reported that their children used Calm when lying down to go to bed (1113/1529, 72.79%). When using Calm at night, children were most likely to use sleep stories (1144/1207, 94.78%), followed by music and soundscapes (749/1114, 67.24%), meditations (736/1120, 65.71%), and breathing exercises (610/1092, 55.86%). Nearly all parents believed that sleep stories were helpful for their children’s sleep (1090/1128, 96.63%), and the majority felt the other components were also helpful (music and soundscapes [570/728, 78.30%], meditations [445/696, 63.94%], breathing exercises [322/576, 55.90%]). Older children used meditations and breathing exercises more often than younger children, and girls used breathing exercises more often than boys (see Table 3).

**Table 1:** Characteristics of parents whose children used Calm.

Characteristic		Participants, n (%)
**Gender**		
	Male	141 (10.14)
	Female	1250 (89.86)
**Ethnicity**		
	Hispanic	89 (6.46)
	Non-Hispanic	1288 (93.54)
**Race**		
	White or European American	1166 (78.31)
	Asian or Asian American	49 (3.29)
	Black or African American	35 (2.35)
	American Indian or Alaskan Native	20 (1.34)
	Hawaiian or Pacific Islander	6 (0.40)
	Biracial or multiracial	58 (3.90)
	Other race	66 (4.43)
**Sleep difficulties**		
	Falling asleep	993 (64.61)
	Staying asleep	631 (41.05)
	Waking up too early	170 (11.06)
	Getting a restful night’s sleep	604 (39.30)
	None	221 (14.38)

**Table 2:** Reasons for starting Calm by age (n=751).^a^

Reason		Coefficient (95% CI)	*P* value
**Improve sleep**			
	Constant	5.73	<.001
	Age	0.93 (0.90-0.97)	<.001
**Reduce stress**			
	Constant	0.09	<.001
	Age	1.18 (1.13-1.22)	<.001
**Reduce depression or anxiety**			
	Constant	0.64	<.001
	Age	1.20 (1.16-1.26)	<.001
**Improve overall health**			
	Constant	0.11	<.001
	Age	1.01 (0.96-1.06)	.82

^a^In all models, coefficients for constants reflect intercepts in the logistic regression models, which can be interpreted as the odds that parents endorsed the outcome (reason for using Calm) when the predictor (age) is equal to 0. Age was a continuous variable.

**Table 3:** Frequency of component usage by age and gender.^a^

Model			Coefficient		SE		*P* value	
**Component usage by age (n=751)**								
	**Sleep stories**							
		Constant		3.58		0.26		<.001
		Age		–0.4		0.03		.17
	**Music**							
		Constant		2.56		0.30		<.001
		Age		–0.03		0.03		.29
	**Meditations**							
		Constant		0.57		0.25		.02
		Age		0.09		0.03		<.001
	**Breathing exercises**							
		Constant		0.38		0.25		.13
		Age		0.09		0.02		.001
**Component usage by gender (n=717)**								
	**Sleep stories**							
		Constant		3.14		0.16		<.001
		Gender=female		0.28		0.22		.20
	**Music**							
		Constant		2.25		0.20		<.001
		Gender=female		0.05		0.26		.84
	**Meditations**							
		Constant		1.29		0.16		<.001
		Gender=female		0.26		0.21		.23
	**Breathing exercises**							
		Constant		0.91		0.16		<.001
		Gender=female		0.48		0.22		.03

^a^In all models, coefficients for constants reflect intercepts in the regression models, which can be interpreted as the expected values of the outcome (weekly usage frequency) when the predictor (age, gender) is equal to 0. Age was a continuous variable. Gender was a dichotomous variable in which male was coded as 0, and female was coded as 1 (therefore, the intercept can be interpreted as the expected value for boys).

## Discussion

### Principal Findings

To our knowledge, this is the first study to assess children’s engagement with the popular mindfulness app Calm and the potential benefits associated with its use. Most children who used Calm were elementary school aged (ie, 4 to 11 years), with similar rates of use in boys and girls. Older children were more likely to use Calm to reduce stress, anxiety, or depression compared with younger children, who were more likely to use Calm for better sleep. According to parents, children mostly used Calm at night, with sleep stories being the most popular component and the component perceived as the most helpful for their child’s sleep.

Parents reported similar rates of app usage for boys and girls, but girls used the app longer (continued to use it with age). Research has shown that in adults, women meditate more, find it more enjoyable, and report greater benefits from it than do men [18]. Given that younger boys appear to be just as likely to meditate as younger girls, mindfulness-based apps may consider content that engages boys and will continue to engage them with age, given their needs and preferences as they get older.

Interestingly, older children and adolescents were more likely to begin using Calm to reduce stress, depression, or anxiety, while younger children were more likely to begin using Calm to improve sleep. Marked increases in stressors have been observed in children and adolescents as they age [19], which may also explain the greater likelihood of older children and adolescents using Calm for their mental health. Additionally, our data suggest that parents reported the usage of Calm in their children to decline around the age of 11 years, which also highlights the need for consumer-based mindfulness mobile apps to better meet the needs of children and adolescents (eg, age-appropriate content, peer support, resources).

Although this app was not exclusively designed for children, Calm provides child-specific content, and many parents use this app. This survey revealed that children use Calm for sleep, and potential positive effects in mental health may be observed. Future research should include randomized controlled trials to test the app’s effectiveness in children.

Finally, as the majority of parents indicated that sleep was a primary reason their children used Calm and that their children used Calm when lying down to go to bed, these data highlight the need for mobile apps to target sleep in children and adolescents. Although sleep stories were appealing and helpful, older children were also likely to use meditations and breathing exercises to help them sleep. This suggests that app developers may want to create content appropriate for a teenage or adolescent audience and that mindfulness-based apps developed for children should incorporate strategies to improve sleep specifically.

### Limitations

Though this was one of the first studies that explored parents’ perceptions of their children’s engagement with a commercially available mindfulness-based app, there are limitations. First, most of our sample was White and well educated. There is a need to explore perceptions in more diverse populations. Second, parents’ perceptions of their children’s engagement in the Calm app may be biased, especially in parents who used Calm for sleep, were generally satisfied with Calm, and reported it to be helpful for their sleep, as they may have perceived Calm to be more helpful compared with what their children believed. However, studies suggest that children, especially those younger than 7, are not able to respond accurately to self-reported behavior, and parents’ perceptions may be more useful data [20]. Although children in this sample were older than 7, age was unknown before the survey was administered. Third, this was a cross-sectional survey, and therefore, we do not know the true effects of the Calm app for improving sleep in children.

### Conclusions

This is the first study to explore how children and adolescents use consumer-based mindfulness mobile apps. These data are important, as they suggest that children, especially younger children, and adolescents of parents who engage in a mindfulness-based apps often use these apps for sleep and that as children get older, they are more likely to use these apps to for their mental health. However, more research is needed to confirm the feasibility and effectiveness of these apps for improving sleep and mental health in children and adolescents.
